# Biological Effect of Mycosporine-Gly-Ser (Shinorine) Against Bis-Retinoid *N*-Retinyl-*N*-Retinylidene Ethanolamine- and Blue-Light-Induced Retinal Pigment Epithelium Cell Damage

**DOI:** 10.3390/nu17081363

**Published:** 2025-04-16

**Authors:** Seung-Yub Song, Jeong-Yong Cho, Dae-Hun Park, Si-Hun Song, Sung-Ho Lee, Jin-Woo Park, Han-Kyu Lim, Seung-Sik Cho

**Affiliations:** 1 Department of Pharmacy, College of Pharmacy, Mokpo National University, Muan 58554, Jeonnam, Republic of Korea; 2 Biomedicine, Health & Life Convergence Sciences, BK21 Four, College of Pharmacy, Mokpo National University, Muan-gun 58554, Jeonnam, Republic of Korea; 3 Department of Integrative Food, Bioscience and Biotechnology, Chonnam National University, Gwangju 61186, Jeollanam-do, Republic of Korea; 4 College of Oriental Medicine, Dongshin University, Naju-si 58245, Jeonnam, Republic of Korea; dhj1221@hanmail.net; 5 Department of Marine and Fisheries Resources, Mokpo National University, Muan 58554, Jeonnam, Republic of Korea

**Keywords:** mycosporine-like amino acid, black paper, *Porphyra dentata*, eye health, ARPE-19

## Abstract

Shinorine is a mycosporine-like amino acid isolated from laver (*Porphyra dentata*), and interest in its functionality has increased recently due to increased production using yeast. There have been few reports on the pharmacological activity of shinorine, and we sought to find the pharmacological significance of shinorine. In the present study, we investigated the pharmacological effects of shinorine purified from *Porphyra dentata* on ARPE-19 cells. First, when ARPE-19 cells were treated with bis-retinoid *N*-retinyl-*N*-retinylidene ethanolamine (A2E) and blue light (BL), cytotoxicity increased, and apoptosis was observed. We investigated the effects of shinorine on A2E- and BL-induced cytotoxicity and changes in apoptotic factors, inflammation, and carbonyl stress. A2E and BL exposure increased ARPE-19 cell apoptosis, but this increase was attenuated by shinorine in a concentration-dependent manner. Treatment with A2E and BL induced ARPE-19 cell apoptosis, but treatment with shinorine decreased the apoptotic factors, such as MAPKs. Shinorine reduced *p*-JNK and *p*-P38, which were increased by A2E and BL. In addition, shinorine was found to regulate inflammatory proteins and proteins associated with carbonyl stress. In conclusion, shinorine may suppress cell damage caused by A2E treatment and BL exposure at the cellular level by regulating various cell death and inflammatory response pathways.

## 1. Introduction

Mycosporine-like amino acids (MAAs) are metabolites derived by various marine organisms. In particular, MAAs are secondary metabolites to protect against light, such as ultraviolet (UV) light [1]. Microorganisms, such as cyanobacteria and *Microcystis aeruginosa*, produce MAAs, such as asterina-330 and shinorine, for photoprotection [2,3].

MAAs typically contain cyclohexenone or cyclohexenimine bonded to one or two amino acids or alcohols at the C1 and C3 positions [4]. To date, approximately 35 MAAs have been identified. One biological effect of MAA is its UV-blocking capability [5].

Recently, MAAs were reported to exert various biological effects. Shinorine is an MAA that is used for its varying biological effects. According to Fernandes et al., shinorine, porphyra-334, and mycosporine-glycine exhibit low toxicity in mouse fibroblasts [6]. Porphyra-334 and shinorine have also been reported to alleviate oxidative stress caused by ultraviolet rays by regulating the Nrf2 pathway [7]. Becker et al. revealed that shinorine and porphyra-334 affect the NF-κB pathway in vitro [8]. Shinorine and porphyra-334 regulate genes involved in inflammation, oxidative stress, and skin aging [9]. Recently, some MAAs have been reported to exhibit anticancer and wound-healing effects. Therefore, besides alleviating UV-induced damage, MAAs may be used in different pharmaceutical applications [10,11].

Shinorine is found in *Chordata*, *Cyanobacteria*, *Dinofluagellata*, *Lichen*, *Miozoa*, *Mollusca*, *Ochrophyta*, *Phaeophyta*, *Porifera*, and *Rhodophyta* [12,13]. Recently, 68 mg/L of shinorine were produced by manipulating a *Saccharomyces cerevisiae* strain. Therefore, further studies must be conducted to confirm the various functions and commercial applications of shinorine [14].

We found no studies on the effects of shinorine on eye health. The importance of eye protection due to consistent use of electronic devices is increasing. Excessive use of video equipment was found to increase the risk of macular degeneration and dry eye syndrome [15]. Long-term exposure to electronic devices has been linked to age-related macular degeneration (AMD) [16]. The BL from cellular phones comprises visible light ranging from 380 to 500 nm. Upon energy accumulation, substantial damage to retinal cells is induced [17]. Short wavelengths can increase intracellular free radical levels and induce cell damage. Exposure to short wavelengths is a known risk factor for uveal melanoma.

In our experiment, we aimed to investigate the preventive effect of shinorine against eye diseases and its effect on ocular cell damage induced by blue light (BL) and ultraviolet rays.

In a previous report, BL caused oxidative stress in dry AMD. BL has been reported to cause mitochondrial damage, such as weakening of the retinal layer and retinal cell death. In particular, mitochondrial functions were reported to deteriorate [18].

In the present study, shinorine at micromolar levels was found to efficiently reduce retinal pigment epithelial (RPE) cell damage caused by BL and reactive oxidants. In addition, the mechanism of action of shinorine was confirmed at the cellular level. Collectively, the results provide evidence supporting the development of shinorine as an effective functional material for the curation/prevention of macular disease.

## 2. Materials and Methods

### 2.1. Cytotoxocity

ARPE-19 cells (retinal pigment epithelium cells, from human) were used in vitro (ATCC, Manassas, VA, USA). The cells were maintained with Dulbecco’s Modified Eagle’s Medium containing 10% fetal bovine serum. The cells were cultured at 37 °C with 5% CO_2_.

Shinorine was isolated according to a previously reported method. Shinorine was purified from laver by organic solvent extraction, RP-18 silica gel column, and Prep-LC, and the purity was 100%. The molecular structure was determined by NMR [19]. The ARPE-19 cells were treated with lutein and shinorine. Cell damage was induced using A2E (10 μM) and BL (20 mW/cm^2^, 15 min). The ARPE-19 cells were seeded in a 96-well plate (5 × 10^3^ cells/well). Then, the cells were continuously treated with lutein (10 μM) and shinorine (1.25–5 μM) for 24 h, and an MTT assay was performed according to previous reference [20]. When evaluating cytotoxicity, the lutein showed no toxicity below 10 μM, and the shinorine also showed no toxicity below 5 μM. Therefore, the maximum treatment concentration of the test substance was set at 10.5 μM.

### 2.2. Fluorescence-Activated Cell-Sorting (FACS) Assay

We aimed to determine the ratio of live cells and apoptotic cells after treatment with A2E and BL and the cellular patterns that changed after treatment with lutein and shinorine.

The ARPE-19 cells were seeded in a 6-well plate (2 × 10^5^ cells/well) for 24 h. The treatment substances were shinorine and lutein, and shinorine was treated in the range of 1.25–5 μM and lutein at 10 μM. The cells were treated with A2E (10 μM) and then exposed to BL (20 mW/cm^2^) for 15 min. After 24 h, the cells were washed with PBS, harvested using trypsin, and centrifuged. The cells were suspended with binding buffer and were treated with Alexa Fluor 488 annexin V (5 μL) and PI (1 mg/mL, 1 μL). A Guava easyCyte instrument (Millipore, Burlington, MA, USA) was used for cell sorting.

### 2.3. TUNEL Assay

The ARPE-19 cells were seeded at a density of 1 × 10^4^ cells/well in a 4-chamber apparatus. After 24 h, the medium was removed, and the cells were treated with shinorine (1.25–5 μM) and lutein (10 μM). Then, the cells were co-treated with A2E and BL. Following medium removal, the cells were fixed with 4% formaldehyde and incubated with 0.25% Triton X-100. The cells were treated with TdT for one hour at 37 °C. Following nuclear staining with DAPI, a confocal microscope was used for imaging.

### 2.4. Western Blot

The ARPE-19 cells were co-treated with A2E and BL exposure. The cells were harvested using protease inhibitors and an RIPA buffer, and then, they were centrifuged for 20 min. The proteins were transferred to PVDF membranes and incubated with 5% skim milk for 2 h at 4 °C and then incubated with primary antibodies (1/100–1/500 dilution) overnight at 4 °C. Bcl-xL, Bcl-2, and GADPH were purchased from Invitrogen, and Bax and Bim were purchased from Santa Cruz Biotechnology. *p*-JNK, JNK, and *p*-P38 were purchased from Cell Signaling Technology. *p*-NF-κB, NF-κB, *p*-IκBα, and IκBα were purchased from Thermo Fisher Scientific Inc. (Waltham, MA, USA). PGE_2_, COX-2, 4-HNE, and malondialdehyde were purchased from Abcam. The membrane was incubated with secondary antibodies (~1/200 dilution).

### 2.5. ELISA Analysis

Human ELISA kits for interleukin tumor necrosis factor alpha (TNF-α) and interleukin-6 (IL-6) and 1-beta (IL-1β) were purchased from the Thermo Fisher Scientific corporation. The samples were incubated with capture antibodies overnight at 4 °C. Following three washes with 0.05% Tween-20, the standards and samples were incubated for 2 h at 4 °C, followed by 1 h at room temperature. Following the addition of a stop solution, the sample was measured at 450 nm.

### 2.6. Immunofluorescence (IF) Analysis

The levels of *p*-NF-κB and COX-2 were determined via immunofluorescence analysis. The ARPE-19 cells were seeded at 1 × 10^4^ cells/well in a 4-chamber apparatus. After 24 h, the medium was removed, and the cells were treated with shinorine (1.25–5 μM) and lutein (10 μM). The cells were treated with A2E (10 μM) after 24 h and exposed to BL (20 mW/cm^2^) for 15 min. The cells were washed with PBS, fixed with 4% formaldehyde, and incubated with Triton X-100 (Thermo Fisher Scientific Inc.). The cells were blocked using 1% bovine serum albumin (BSA), incubated with primary antibodies overnight at 4 °C, washed thrice with PBS, and incubated with secondary antibodies. Following nuclear staining with DAPI, *p*-NF-Κb, COX-2, Alexa Fluor 488-conjugated anti-rabbit IgG, or Alexa Fluor 555-conjugated anti-goat IgG was employed.

### 2.7. Statistical Analysis

The experimental results are expressed as the mean ± standard deviation (SD). Statistical significance was determined using one-way analysis of variance (ANOVA) and Dunnett’s multiple comparison test. Excel software was used. The *p* value was set at *p* < 0.05. All the tests were repeated three times.

## 3. Results and Discussion

### 3.1. Cytotoxicity

**Figure 1:**
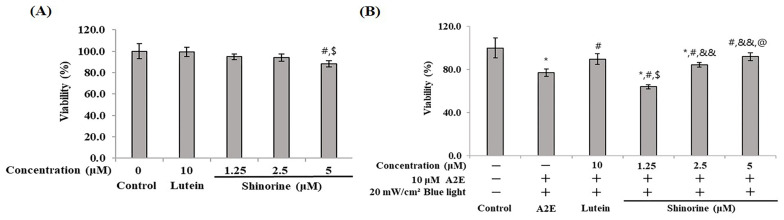
Cytotoxicity of shinorine on ARPE-19. (**A**) ARPE-19 cells treated with lutein (10 µM) and shinorine (1.25–5 µM); (**B**) shinorin attenuated A2E- and BL-induced cytotoxicity. Mean ± S.D. (*n* = 3) triplicate. * *p* < 0.05 vs. CON; ^#^ *p* < 0.05 vs. A2E; ^$^ *p* < 0.05 vs. Lutein; ^&&^ *p* < 0.001 vs. 1.25 µM; ^@^ *p* < 0.05 vs. 2.5 µM.

As presented in Figure 1A, lutein and shinorine are considered safe when used at concentrations of up to 5 μM. Thus, subsequent experiments were performed with up to 5 μM of these compounds. Cytotoxicity was found to be reduced due to the A2E and BL doses dependently. The cell survival rate was approximately 76% following treatment with A2E and BL. Treatment with 10 μM of lutein resulted in a cell survival rate of 89%, while treatment with 5 μM of shinorine resulted in a survival rate of 92% (Figure 1B).

### 3.2. Shinorine Regulated the Apoptotic Factors in ARPE-19

**Figure 2:**
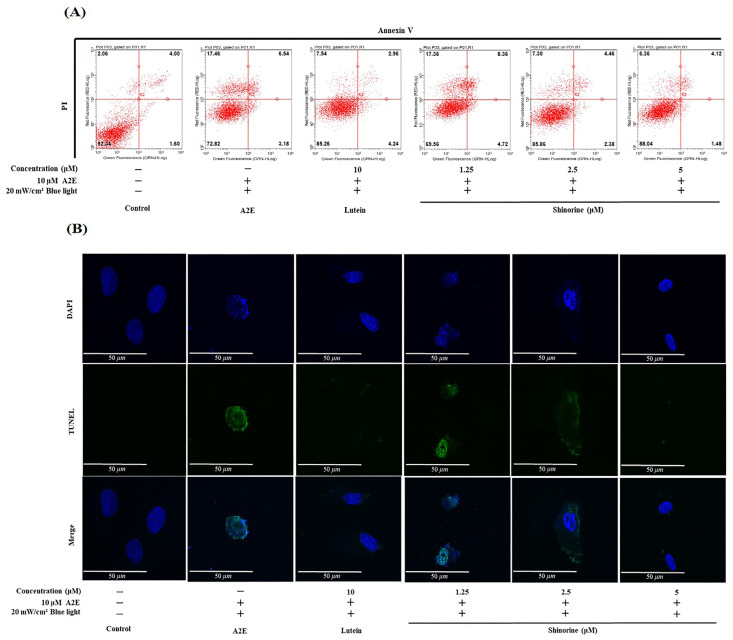
Shinorine reduces apoptosis induced by A2E and BL. (**A**) Annexin V/PI staining and flow cytometry. Q1: Necroptotic cells; Q2: Late apoptotic cells; Q3: Early apoptosis; Q4: Live cells. (**B**) TUNEL results indicating a decrease in apoptosis with an increase in shinorine concentration. α-tubulin (green) and DAPI (blue) were visualized using a confocal microscope. Magnification 10×; Scale bar, 50 μm.

When A2E and BL were simultaneously administered, the number of apoptotic cells increased in the order of 10% (Figure 2). In the lutein treatment group, the apoptotic cells decreased to approximately 6.7%. Shinorine reduced the number of apoptotic cells in a concentration-dependent manner (Figure 2A). In fact, 5 µM of shinorine decreased the number of apoptotic cells to approximately 5.8%, which aligns with the percentage of apoptotic cells found in the untreated group. As shown in Figure 2B, in particular, shinorine also reduced A2E- and BL-induced cell death in a concentration-dependent manner.

**Figure 3:**
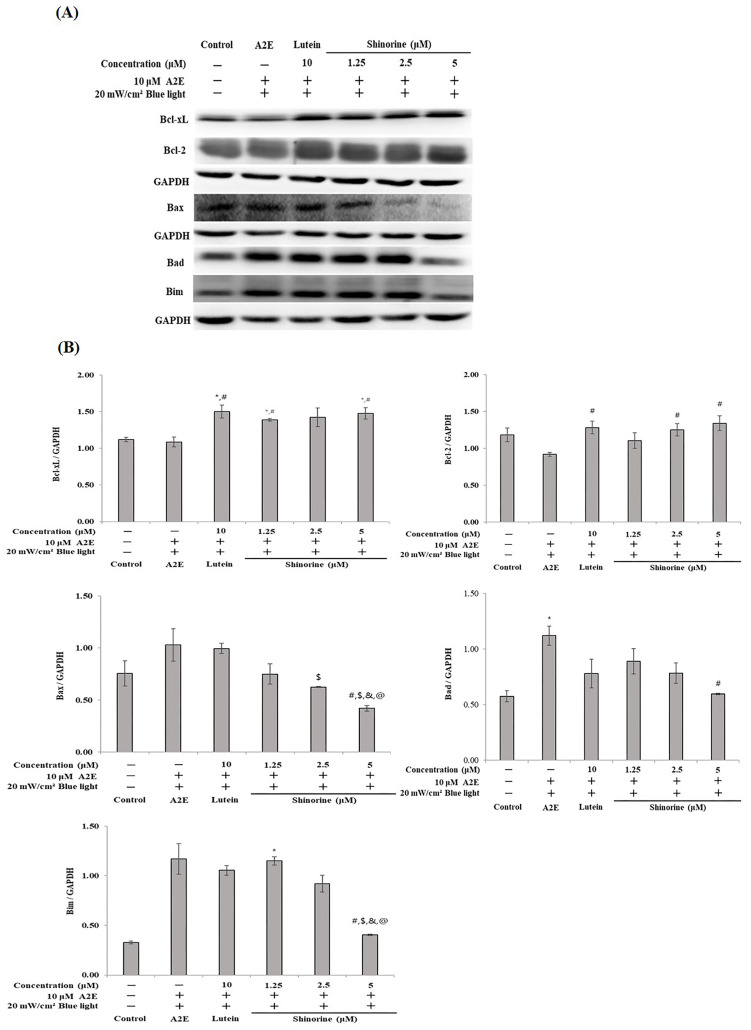
Expression of Bcl family induced by A2E plus BL. (**A**) Western blot of Bcl proteins. (**B**) Densitometry (Image J 1.54 software) of expressed Bcl proteins. Mean ± S.D. (*n* = 3), triplicate. * *p* < 0.05 vs. CON; ^#^ *p* < 0.05 vs. A2E; ^$^ *p* < 0.05 vs. Lutein; ^&^ *p* < 0.05 vs. 1.25 µM; ^@^ *p* < 0.05 vs. 2.5 µM.

Figure 3 shows the expression patterns of apoptosis. When A2E and BL were co-treated, the expression of Bcl-2 decreased. However, in the groups treated with 10 µM lutein and 5 µM shinorine, the Bcl-2 expression was almost restored to the control level. The Bcl-xL expression did not reveal significant changes following treatment with A2E and BL; however, treatment with lutein and shinorine increased the expression of Bcl-xL. In terms of Bax and Bim, lutein (10 µM) could not reduce the expression induced by A2E and BL; however, shinorine reduced the expression of Bax, Bad, and Bim.

### 3.3. Shinorine Regulated the p38 and JNK in ARPE-19

**Figure 4:**
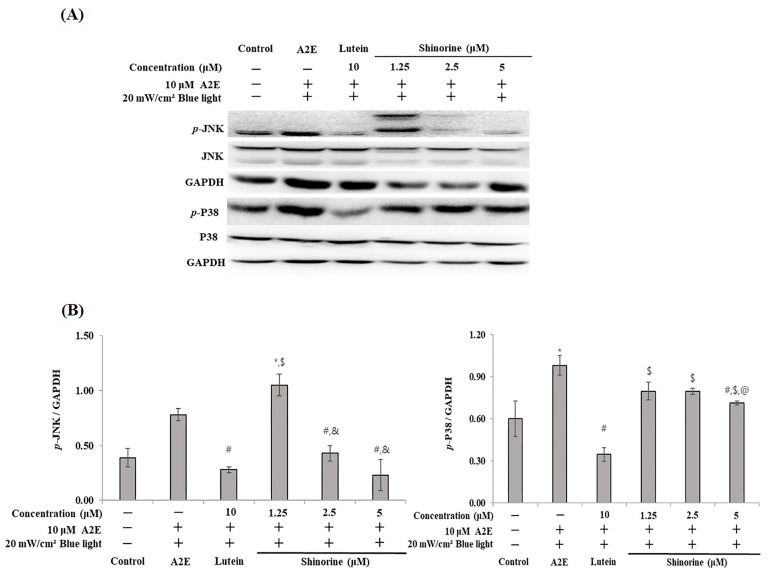
Expression of of MAPKs induced by A2E plus BL. (**A**) Western blots of *p*-JNK, JNK, *p*-P38, P38, and GAPDH. (**B**) Densitometry (Image J software) of expressed MAPKs. Mean ± S.D (*n* = 3). Triplicate, * *p* < 0.05 vs. CON; ^#^ *p* < 0.05 vs. A2E; ^$^ *p* < 0.05 vs. Lutein; ^&^ *p* < 0.05 vs. 1.25 µm; ^@^ *p* < 0.05 vs. 2.5 µM.

As shown in Figure 4, by determining the expression of MAPKs related to apoptosis, the phosphorylation levels of JNK and p38 were found to increase following the simultaneous administration of A2E and BL (Figure 4A). Notably, shinorine reduced the expression of *p*-JNK and weakly inhibited that of *p*-p38 in a dose-dependent manner. Compared to lutein (10 µM), shinorine (5 µM) exhibited a marked inhibition of *p*-JNK expression. Treatment with lutein markedly inhibited the expression of *p*-p38 (Figure 4B).

### 3.4. Shinorine Regulated the Inflammatory Response in ARPE-19 Cells

**Figure 5:**
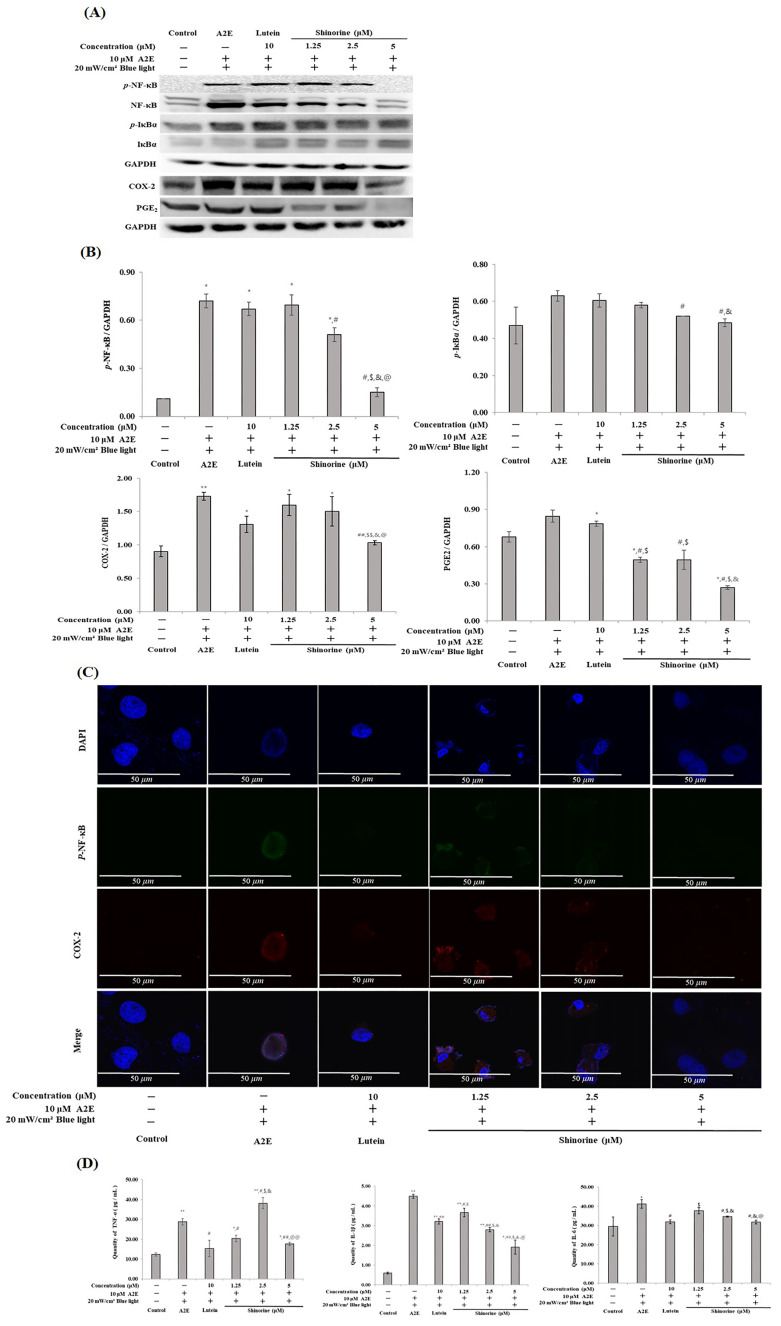
Shinorine has anti-inflammatory effect in ARPE-19. (**A**) Expression of inflammatory biomarkers; (**B**) densitometry (Image J software) of expressed inflammatory biomarkers; (**C**) immunofluorescence images of *p*-NF-κB and COX-2. DAPI (blue) was visualized using a confocal microscope. Magnification 10×; Scale bar, 50 μm; (**D**) ELISA assay of pro-inflammatory cytokines. Mean ± S.D. (*n* = 3). * *p* < 0.05 vs. CON; ** *p* < 0.001 vs. CON; ^#^ *p* < 0.05 vs. A2E; ^##^ *p* < 0.001 vs. A2E; ^$^ *p* < 0.05 vs. Lutein; ^$$^ *p* < 0.001 vs. Lutein; ^&^ *p* < 0.05 vs. 1.25 µM; ^@^ *p* < 0.05 vs. 2.5 µM; ^@@^ *p* < 0.001 vs. 2.5 µM.

Shinorine was effective in regulating the inflammatory proteins that were increased by the A2E and BL treatments. As shown in Figure 5A–C, Shinorine reduced the expression levels of *p*-NF-kB, COX-2, and PGE2 that were increased by A2E and BL. In addition, shinorine regulated the production of pro-inflammatory cytokines (Figure 5D).

### 3.5. Shinorine Regulated the Carbonyl Stress Induced by A2E and BL

**Figure 6:**
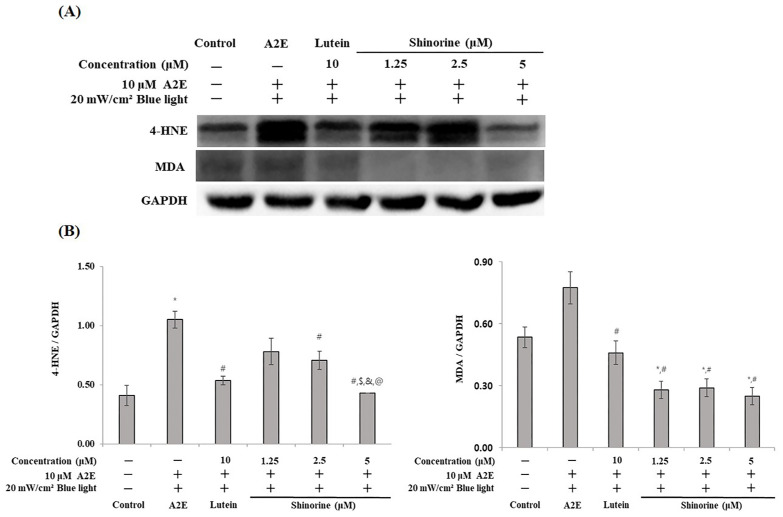
Shinorine decreases carbonyl stress induced by BL plus A2E. (**A**) Expression of 4-HNE, MDA, and GAPDH; (**B**) densitometry (Image J software) of 4-HNE, MDA. Mean ± S.D. (*n* = 3). * *p* < 0.05 vs. CON; ^#^ *p* < 0.05 vs. A2E; ^$^ *p* < 0.05 vs. Lutein; ^&^ *p* < 0.05 vs. 1.25 µM; ^@^ *p* < 0.05 vs. 2.5 µM.

We investigated whether A2E- and BL-induced apoptosis and inflammation were associated with carbonyl stress. Shinorine decreased the expression levels of 4-HNE and MDA, which were induced by A2E and BL (Figure 6A,B).

## 4. Discussion

In the present study, treatment with A2E and BL was found to induce cytotoxicity in RPE cells. Lutein and shinorine regulated increased inflammatory and apoptotic biomarkers induced by A2E and BL.

BL has been reported to cause damage to eye cells and is linked to diseases such as macular degeneration [21]. In a previous report, cell damage was induced by treatment with A2E and BL. BL causes extreme oxidative stress in dry AMD. In vivo, BL was found to induce apoptosis in the retinal layer and reduce mitochondrial function. In particular, mitochondrial function was found to be clearly weakened by long-term BL exposure [21].

As MAAs, such as shinorine, protect against UV-induced damage [14], we decided to determine whether MAAs can inhibit UV and other types of light-induced damage. We confirmed that 5 µM of shinorine could regulate A2E-induced and BL-induced apoptosis and inflammation in RPE cells. Lutein is a representative eye drug that is broadly used as an ingredient in functional foods and is broadly used worldwide to prevent retinal degeneration [22]. The protective effects of lutein have been demonstrated using RPE cells and mouse models [23]. Therefore, lutein was employed as a control. In this study, 10 µM of lutein were found to alleviate RPE cell damage.

Studies have revealed the UV-protective and wound-healing effect of shinorine [11,14]. To our knowledge, we are the first to report the protective effect of MMA(shinorine) relative to the eye. According to previous studies, MAA is involved in DNA damage, the inhibition of reactive oxygen species (ROS) production, and thymine dimer formation [24]. The activity of MAAs may be based on their respective residue. By comparing shinorine and porphyra-334 residues, shinorine was found to contain serine residue, whereas porphyra-334 exhibited threonine residue. In our preliminary study, when the same concentrations of shinorine and porphyra-334 were administered to cells that were subsequently exposed to A2E and BL, we confirmed that shinorine further reduced the number of apoptotic cells. Therefore, we speculate that serine residue is responsible for the biological difference between shinorine and porphyra-334.

In a previous paper, BL was reported to increase the production of oxi-A2E in RPE cells treated with A2E and induce apoptosis and inflammation. As a result, photoreceptor cells are damaged by lipofuscin and die [25]. Therefore, we sought to elucidate how MAAs such as shinorine protect against inflammation and/or apoptosis of RPE cells induced by A2E and BL. As shown in Figure 1, lutein (10 µM) and shinorine (5 µM) attenuated A2E- and BL-induced cytotoxicity. Therefore, subsequent experiments were performed using concentrations less than 10 µM and 5 µM, respectively (Figure 1).

MAPKs are widely studied and are associated with various processes and diseases, such as inflammation and cancer [26]. MAPKs are closely related to RPE cell differentiation, and they seem to be particularly associated with p38 and JNK [27].

We found that A2E and BL induced the phosphorylation of p38 and JNK in RPE cells, suggesting that apoptosis and inflammatory responses may be induced. UV has an effect on ocular cells, and our results are similar to studies that report that UV increases the expression of ERK1/2, p38, and JNK in RPE cells [28]. Therefore, it can be observed that MAAs such as shinorine regulate ARPE-19 cell damage induced by A2E and BL by regulating the MAPK pathway.

Tsao et al. revealed that the death of RPE cells treated with H_2_O_2_ does not involve ERK1/2 but is affected by JNK and p38 activation [29]. According to Sawyer et al., the activation of ERK1/2 induces apoptosis [30]. In our results, A2E and BL induced p38 and JNK expression, aligning with the results of Tsao et al. Shinorine downregulated A2E-induced and BL-induced JNK activation but only weakly regulated p38 (Figure 4).

Bcl-xL and BCL-2 are apoptotic factors, and cytochrome C is closely related to the BCL family [31]. Treatment with 5 µM of shinorine induced an increase in Bcl-xL and Bcl-2 expression, similarly to that obtained following treatment with 10 µM of lutein. In addition, the expression levels of Bax, Bad, and Bim, which are pro-apoptotic protein-induced by A2E and BL, were suppressed by lutein and shinorine. Such findings align with that of a previous study, in which factors, such as Bad, were found to form dimers with Bcl-xL. Taken together, shinorine inhibits RPE cell death by controlling the apoptotic pathway (Figure 3).

BL has been reported to induce oxidized A2E and ROS production [32]. ROS are known to induce cell death by causing oxidative stress. [33]. Wang et al. described that oxidative stress-induced inflammation is also closely associated with apoptosis of RPE cells [34].

Continuously exposing RPE cells to oxidative stress can damage the mitochondria [35], which eventually worsens macular degeneration. Thus, RPE damage induced by oxidative stress is considered one of the main factors of eye diseases. Fiorani et al. revealed that light exposure activates microglia and induces an inflammatory response. In a similar study, Guillonneau reported that if microglia activate and accumulate in the subretinal space, activated cells release inflammatory cytokines [36]. Therefore, the simultaneous treatment of RPE cells with A2E and BL may be an important factor in inducing ocular diseases through apoptosis and inflammatory responses.

Few reports have been described regarding the mechanisms of action between inflammation and carbonyl/oxidative stress in RPE cells. Yang et al. reported that 4-HNE induces the production of inflammatory cytokines and modified the HSP70 in RPE cells [37].

In the present study, A2E and BL treatments resulted in the increased expression of 4-HNE and MDA; however, treatment with shinorine regulated this effect (Figure 6). This result is associated with the data presented in Figure 5, in which the levels of pro-inflammatory cytokines that were increased following treatments with A2E and BL were downregulated by treatments with shinorine. Thus, it was found that shinorine effectively attenuates the apoptosis and inflammatory response of ARPE-19 cells induced by A2E and BL and is considered an appropriate functional material for the treatment of AMD.

## 5. Conclusions

In the present study, we obtained shinorine from the main component of *Porphyra dentata* in order to verify its pharmacological effect in RPE cells. It was confirmed that apoptosis and inflammatory responses induced by A2E and BL are regulated by shinorine. In addition, apoptosis and inflammatory reactions were found to be associated with carbonyl stress. The results of our study suggest that *Porphyra dentata* and its major constituent, shinorine, have a curative effect on eye health and/or disease, and it is thought that future studies on the safety of shinorine should be combined with in vivo studies.

## Data Availability

Should any raw data files be needed, they are available from the corresponding author upon reasonable request.
